# Detection of 16S rRNA and KPC Genes from Complex Matrix Utilizing a Molecular Inversion Probe Assay for Next-Generation Sequencing

**DOI:** 10.1038/s41598-018-19501-z

**Published:** 2018-02-01

**Authors:** Christopher P. Stefan, Adrienne T. Hall, Timothy D. Minogue

**Affiliations:** United States Army Medical Research Institute of Infectious Disease, Diagnostic Systems Division, Fort Detrick, Maryland, 21702 United States of America

## Abstract

Targeted sequencing promises to bring next-generation sequencing (NGS) into routine clinical use for infectious disease diagnostics. In this context, upfront processing techniques, including pathogen signature enrichment, must amplify multiple targets of interest for NGS to be relevant when applied to patient samples with limited volumes. Here, we demonstrate an optimized molecular inversion probe (MIP) assay targeting multiple variable regions within the 16S ribosomal gene for the identification of biothreat and ESKAPE pathogens in a process that significantly reduces complexity, labor, and processing time. Probes targeting the *Klebsiella pneumoniae* carbapenemase (KPC) antibiotic resistance (AR) gene were also included to demonstrate the ability to concurrently identify etiologic agent and ascertain valuable secondary genetic information. Our assay captured gene sequences in 100% of mock clinical samples prepared from flagged positive blood culture bottles. Using a simplified processing and adjudication method for mapped sequencing reads, genus and species level concordance was 100% and 80%, respectively. In addition, sensitivity and specificity for KPC gene detection was 100%. Our MIP assay produced sequenceable amplicons for the identification of etiologic agents and the detection of AR genes directly from blood culture bottles in a simplified single tube assay.

## Introduction

Molecular diagnostic techniques are common in the clinical setting, co-existing with traditional culture methods for routine pathogen detection and identification^1,2^. The rapid detection of bacterial infections from primary samples using real-time PCR assays based on conserved regions within 16S rRNA shows promise; however, sensitivity issues caused by loss of material during sample processing and high false positives from background contamination result in continued reliance on culture techniques^3,4^. A diagnostic assay with high sensitivity is essential for blood stream infections (BSIs) where bacterial load is often less than 100 CFU/ml^5^. The rise in nosocomial bloodstream infections caused by antibiotic resistant (AR) organisms is of particular concern as this can result in inadequate antimicrobial-therapy and extremely high mortality rates^6^. Typically for full identification after blood culture, several days are necessary for subculture on selective media, gram staining, morphological analysis, and biochemical tests. Further species-specific biochemical tests along with AR testing can further prolong the diagnostic timeframe^7^. This makes diagnostic tests that are able to detect, identify, and characterize an etiologic agent directly from blood culture a valuable tool in the diagnostic arsenal.

The use of agnostic sequencing from primary samples can significantly reduce the diagnostic timeframe compared to culture while also providing etiologic agent identification and secondary genetic information such as the presence of AR genes. This, however, still requires deep sequencing due to a dominant presence of host genome, resulting in low-throughput and high costs. Targeted sequencing focusing on classifying organisms utilizing the bacterial 16S gene addresses this issue. Since the 1970’s, the 16S gene sequence has been used to identify and classify bacterial organisms down to the genus and species level^8^. Initially, use of the entire 1.5 kb target was necessary for taxonomic classification and were obtained using low-throughput Sanger sequencing technologies. In depth analysis of the 16S sequence revealed that only specific hypervariable regions, in particular variable regions V2, V3, and V6 together, were required for identification of common bacterial pathogens^9,10^. Unfortunately, NGS platforms such as Illumina produce read lengths smaller than those required for full length 16S gene sequencing or for amplicons that include the number of variable regions required for low-level taxonomic resolution^10,11^. However, given the sequencing depth afforded by NGS, a multiplexed targeted assay could capture and amplify multiple, short length, hypervariable 16S regions along with AR genes, SNPs, and toxin producing elements for identification and characterization.

Multiple technologies exist for multiplexed upfront target enrichment of DNA samples prior to NGS including PCR, ligation dependent amplification, and hybridization capture (reviewed in^12^). High-level multiplex PCR reactions often result in non-specific amplicons and biased amplification based on the efficiencies of each primer set^12^. Similarly, hybridization/capture techniques require micrograms of input DNA and long hybridization times, which are not conducive to small sample volumes and time-to-answer constraints^13^. Molecular Inversion probes (MIP) are one method to enrich for target sequences using a ligation dependent approach, which allows for a high order of multiplexabiltiy within a single tube reaction^14,15^. MIPs involve target-specific sequence hybridization combined with a “gap-fill” step via polymerase and a subsequent ligation event. These dual enzymatic steps are required for proper capture of the targeted region. Combined with the removal of excess probe and unwanted amplification events via exonuclease reaction, this method allows for high-level multiplexing unattainable with other methods. MIP protocols are more complex and suffer from increased processing times compared to their PCR counterparts. Here, we developed a MIP protocol for sequence capture both decreasing the complexity of the MIP reaction and reducing the time-to-answer. We created a probe pool targeting multiple variable regions within the 16S gene and demonstrate its effectiveness at identifying bacterial organisms using analytical and mock clinical samples. Also demonstrated here is the use of MIPs for pathogen identification and AR detection directly after blood culture.

## Results

### MIP protocol optimization for the 16S probeset

**Figure 1:**
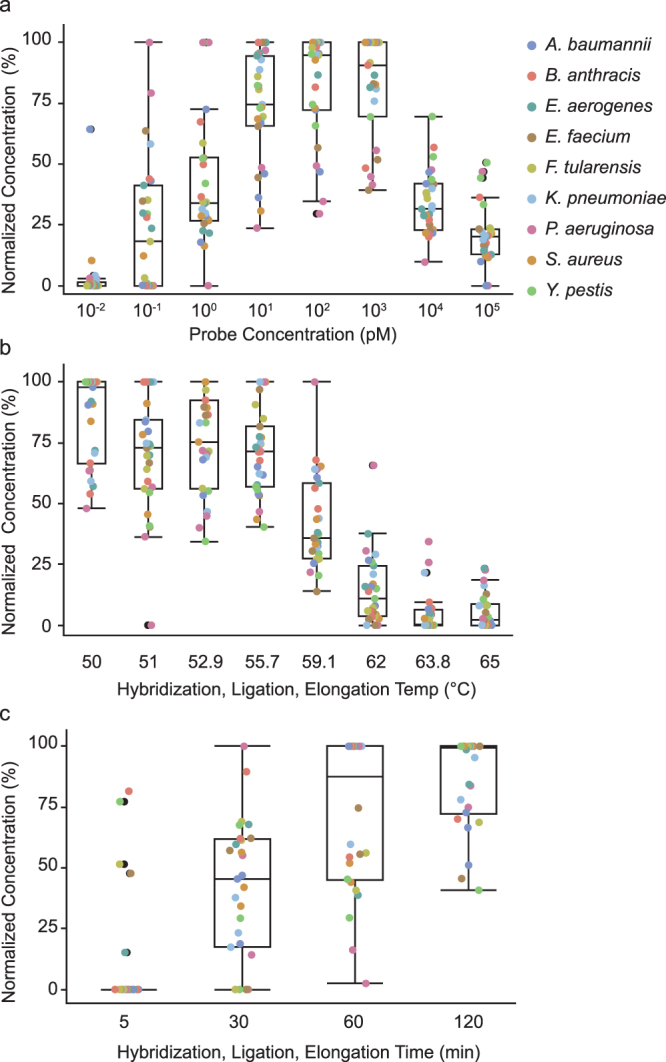
MIP protocol optimization for the 16S probeset. Pooled 16S MIPs were tested against DNA extracts of representative biothreats and ESKAPE pathogens to optimize target capture conditions including (**a**) probe concentration, (**b**) reaction temperature and (**c**) reaction duration. Amplicon formation was quantitatively measured after probe circularization and amplification with the universal primer set across three replicates for each organism at each variable. Concentrations were normalized to 0% and 100% representing the lowest and highest concentrations across the experimental range. Data points for each organism were combined and a corresponding outlier box plot was generated for each variable.

We designed 16S MIP probes (see Supplementary Table S1) to amplify variable regions V1, V2, V3, V6, and V7 of the 16S gene to establish a viable amplification technique for clinical adjudication of bacterial pathogens. These regions are sufficient for classification of most medically relevant bacteria as well as biothreats agents^9^. MIP protocols typically involve four separate steps including an overnight hybridization, “gap-fill” and ligation reactions, an exonuclease step, and captured sequence amplification^14^. We focused on improving the workflow for routine use and decreasing time-to-answer for the 16S probeset. In brief, switching the Stoffel fragment to Phusion polymerase increased processivity and fidelity essential for error-free amplification of 16S regions. We also optimized buffer conditions to reduce high divalent salt concentrations that could impede polymerization^16,17^. To address time-to-answer, we streamlined the protocol by combining the probe hybridization, “gap fill”, and ligation reactions into a single step. We evaluated probe concentrations, reaction temperatures, and reaction times across nine bacterial pathogens (Supplementary Table S2) from diverse phylogeny to determined optimal conditions for amplicon formation (Fig. 1).

Amplicon concentration was measured after amplification with universal primers using a LabChip GX Touch HT. To account for data variances across experiments and bacterial strains, concentrations were normalized to 0% and 100% representing the lowest and highest concentrations across the experimental range. Assessment of probe pool concentrations across 10-fold dilutions (Fig. 1a) showed reactions containing 10, 100, and 1000 pM probe concentrations produced the highest amount of amplicon without sacrificing purity (Supplementary Figure S1). Optimal reaction temperatures for hybridization, polymerization, and ligation enzymes span 72 °C to 45 °C; therefore, combining these processes into a single step required testing across a broad temperature range. A reaction temperature of 60 °C significantly reduced amplicon production; while lower temperatures did not greatly impact product formation (Fig. 1b). Overall, data showed 55 °C was ideal for optimal amplicon formation. For optimal hybridization, “gap fill”, and ligation time, we found an increase in amplicon concentration after 30 minutes that increased with reaction time (Fig. 1c). These optimizations produced sequenceable amplicons from all nine organisms in a reproducible protocol suitable for routine use.

### 16S gene detection from relevant matrix

**Figure 2:**
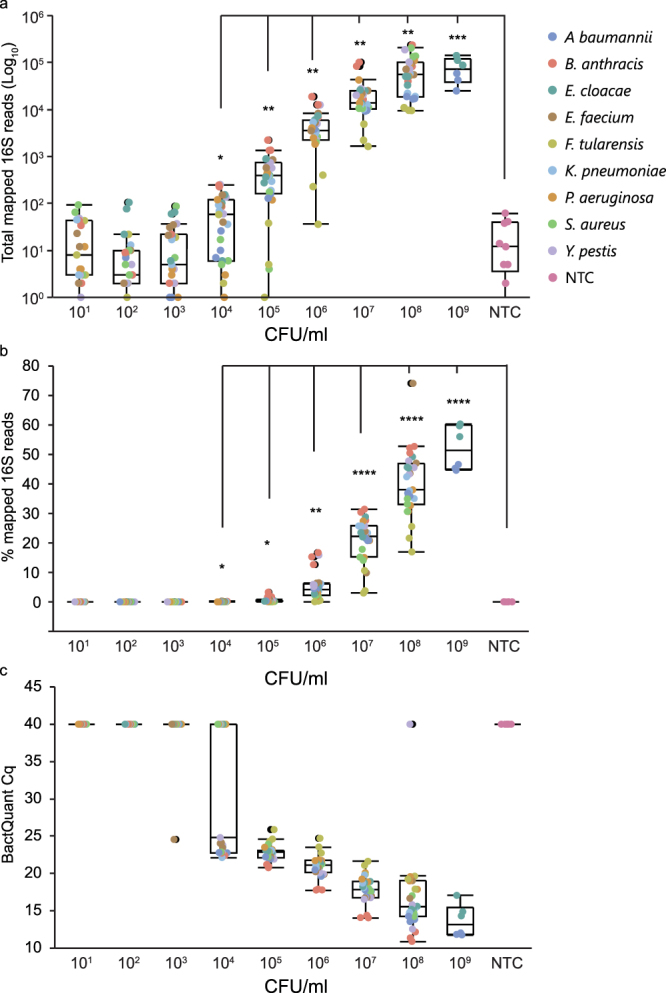
Detection of 16S gene sequence from blood culture matrix utilizing two molecular techniques. Pooled 16S MIPs were tested against DNA extracts prepared from serial-dilutions of blood culture matrix spiked with biothreat and ESKAPE pathogens at concentrations ranging from 10^9^–10^1^ CFUs/ml. Sequencing reads were processed and mapped against three reference databases containing 16S variable regions V1/2, V3, and V6/V7. (**a**) Total mapped sequencing reads (**b**) percentage of mapped sequencing reads and (**c**) C_q_s resulting from real-time PCR with the BactQuant assay is plotted versus CFU/ml. Three independently extracted replicates for each organism are represented. Data points were combined and a corresponding outlier box plot was generated for each variable. Prepared blood culture without pathogen was used as a negative control. Unpaired parametric t-tests were used for statistical evaluation. P values <0.05, 0.01, 0.001, and 0.0001 are indicated with asterisks *, **, ***, and **** respectively.

Automated blood culture serves as the gold standard method to detect bacteremia in patients presumed to have BSIs. In this context, we titrated both ESKAPE and biothreat pathogens in blood culture media at CFUs/ml ranging from 10^9^–10^1^ and tested the sensitivity of the optimized MIP protocol. Statistical significance using an unpaired, non-parametric t-test demonstrated a significant difference compared to non-template control samples in total mapped 16S reads as low as 10^4^ CFU/ml. Notably, only 59% of samples at this concentration had a total number of reads mapping above the non-template control background (median plus 2× standard deviation) (Fig. 2a). In contrast, the samples with total mapped reads above background at 10^5^ and 10^6^ CFU/ml were 88% and 100%, respectively. Two of the failed samples, at 10^5^ CFU/ml, were *F. tularensis*, which yielded significantly less reads at all concentrations tested. To mitigate variances between samples, we normalized total sequencing reads mapped to the total sequencing reads per sample (Fig. 2b). Similar to previous results, 10^4^ CFU/ml showed statistically significant changes compared to non-template controls.

We also tested the BactQuant assay, a 16S TaqMan® quantitative real-time PCR assay^4^, as a representative comparator for other 16S molecular diagnostic techniques (Fig. 2c). In this study, the BactQuant had a limit of detection (LOD), the concentration at which all three replicates were positive, of 10^5^ CFU/ml. At 10^4^ CFU/ml, 51% of the samples fell below a positive threshold C_q_ of 40. These percentages were similar to those seen with the MIP assay, thus demonstrating comparable performance between the two assays. In terms of clinical relevance, these results showed positive detection of both assays within the average CFU/ml, 10^7^–10^8^, seen for a flagged positive culture using the BACTEC FX blood culture system^18^.

### 16S taxonomic classification from relevant matrix

**Figure 3:**
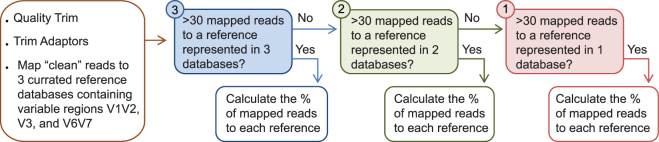
Processing method for taxonomic classification from mapped sequencing reads. Sequencing reads are initially trimmed for quality and adaptors before mapping to three reference databases composed of variable regions V1/V2, V3, and V6/V7. Reference species with greater than 30 reads are grouped into their representative number of databases. The percentage of mapped reads was then calculated and a “best hit” approach was used for final taxonomic classification.

Detection of 16S sequences from blood culture confirms bacterial infection; however, the benefit of sequenced-based diagnostics lies in taxonomical classification of the etiologic agent. The strength of sequencing multiple variable regions lays in the expectation of concordant etiologic agent representation in each variable region, thus reducing false positives. To account for this, we composed three databases composed of variable regions V1 and V2, V3, and V6 and V7 from each reference organism and mapped sequencing reads to each database. We applied a simple data processing method weighted towards reference organisms that had sequencing reads represented in multiple reference databases (Fig. 3). Reference species with less than 30 mapped sequencing reads were filtered. We grouped the remaining references based on representation in each database and calculated the percentage of mapped reads. The highest identity was then used for final taxonomic classification (Table 1). Using this processing method, genus level concordance was 100% for all input organisms and their replicates. For speciation, 100% of the sequencing reads for *A. baumannii, K. pneumoniae, F. tularensis*, and *P. aeruginosa* agreed with the spiked input. *B. anthracis*, *E. faecium*, and *E. cloacae* had multiple species level hits; however, the best hit agreed with expected results. Unsurprisingly, *Y. pestis* and *S. aureus* could not be distinguished from *Y. pseudotuberculosis* and *S. argenteus*, respectively, with approximately 50% of the sequencing reads mapping to each. These results were intuitive as *Y. pestis* and *S. aureus* have near identical 16S sequences to *Y. pseudotuberculosis* and *S. argenteus* and require multiple loci for species level identification^19,20^.

**Table 1:** Taxonomic classification of 16S sequencing reads using a simplified processing method for mapped sequencing reads.

Input Organism	CFU/ml	Replicate	*	Hit 1	%		Hit 2	%		Hit 3	%
*B. anthracis*	10^8^	A	**3**	*B. anthracis*	79.31	**3**	*B. cereus*	20.69	**2**	*B thuringiensis*	35.26
		B	**3**	*B. anthracis*	67.03	**3**	*B. cereus*	19.97	**3**	*B. toyonensis*	6.78
		C	**3**	*B. anthracis*	77.47	**3**	*B. cereus*	22.53	**2**	*B thuringiensis*	50.95
*Y. pestis*	10^8^	A	**3**	*Y. pestis*	52.25	**3**	*Y. pseudotuberculosis*	47.75	**1**	*Y. similis*	53.43
		B	**3**	*Y. pestis*	52.06	**3**	*Y. pseudotuberculosis*	47.94	**2**	*Y. similis*	67.17
		C	**3**	*Y. pestis*	43.64	**3**	*Y. pseudotuberculosis*	38.65	**3**	*Y. similis*	17.71
*F. tularensis*	10^8^	A	**3**	*F. tularensis*	100	**2**	*F. hispaniensis*	100	**1**	*F. persica*	22.37
		B	**3**	*F. tularensis*	100	**2**	*F. hispaniensis*	100	**1**	*F. persica*	22.46
		C	**3**	*F. tularensis*	100	**2**	*F. hispaniensis*	100	**1**	*F. persica*	20.83
*E. cloacae*	10^8^	A	**3**	*E. cloacae*	65.09	**3**	*E. xiangfangensis*	34.91	**2**	*E. asburiae*	58.08
		B	**3**	*E. cloacae*	64.39	**3**	*E. xiangfangensis*	35.61	**2**	*E. asburiae*	56.56
		C	**3**	*E. cloacae*	65.74	**3**	*E. xiangfangensis*	34.26	**2**	*E. asburiae*	57.35
*S. aureus*	10^8^	A	**3**	*S. argenteus*	55.69	**3**	*S. aureus*	44.31	**2**	*S. simiae*	67.70
		B	**3**	*S. argenteus*	57.02	**3**	*S. aureus*	42.98	**2**	*S. simiae*	67.03
		C	**3**	*S. argenteus*	55.40	**3**	*S. aureus*	44.60	**2**	*S. simiae*	37.17
*K. pneumoniae*	10^8^	A	**3**	*K. pneumoniae S000003514*	100	**2**	*K. variicola*	54.66	**2**	*K. pneumoniae S000387414*	26.62
		B	**3**	*K. pneumoniae S000003514*	100	**2**	*K. variicola*	51.63	**2**	*K. pneumoniae S000387414*	28.36
		C	**3**	*K. pneumoniae S000003514*	100	**2**	*K. variicola*	53.41	**2**	*K. pneumoniae S000387414*	27.13
*A. baumannii*	10^8^	A	**3**	*A.baumannii*	100	**1**	*A. venetianus*	31.86	**1**	*A. junii*	31.51
		B	**3**	*A.baumannii*	100	**1**	*A. venetianus*	33.00	**1**	*A. junii*	32.76
		C	**3**	*A.baumannii*	100	**1**	*A. junii*	32.76	**1**	*A. venetianus*	32.38
*P. aeruginosa*	10^8^	A	**3**	*P. aeruginosa*	100	**1**	*P. delhiensis*	8.24	**1**	*P. citronellolis*	8.19
		B	**3**	*P. aeruginosa*	100	**1**	*P. stutzeri*	8.58	**1**	*P. knackmussii*	8.46
		C	**3**	*P. aeruginosa*	100	**1**	*P. stutzeri*	7.44	**1**	*P. knackmussii*	7.41
*E. faecium*	10^8^	A	**3**	*E. faecium*	40.84	**3**	*E. hirae*	23.80	**3**	*E.durans*	17.50
		B	**3**	*E. faecium*	40.80	**3**	*E. hirae*	23.51	**3**	*E.durans*	17.54
		C	**3**	*E. faecium*	39.04	**3**	*E. hirae*	24.03	**3**	*E.durans*	18.24

*Bold indicate number of databases a reference species hit.

### 16S MIP performance with the addition of probes detecting AR genes

Patient outcomes with BSIs are directly correlated with timely antibiotic treatment^6^. Similarly, proper antibiotic stewardship and epidemiological surveillance of acquired resistance genes are vital to mitigate resistance dissemination. In this context, multiplexing capabilities for MIP reactions along with the sequence-specific information afforded by NGS allow detection of multiple targets including the variable regions within the 16S gene and potentially acquired AR genes.

**Figure 4:**
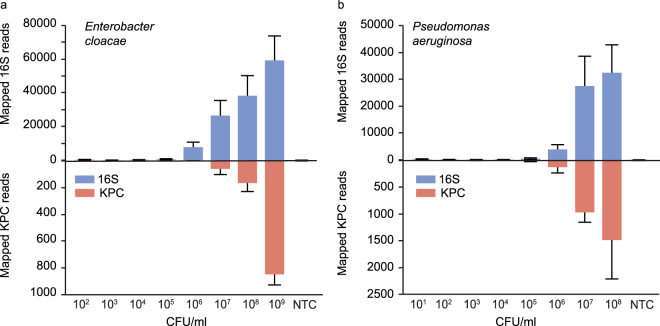
Detection of AR and 16S gene sequences from blood culture matrix. Pooled 16S and KPC MIPs were tested against DNA extracts prepared from serial-dilutions of blood culture matrix spiked with (**a**) *Enterobacter cloacae* and (**b**) *Pseudomonas aeruginosa* at concentrations ranging from 10^9^–10^1^ CFUs/ml. Total reads mapped to 16S references, upper Y-axis, and KPC references, lower Y-axis, are shown for each dilution. Error bars represent the standard deviation of three independently extracted samples.

We designed two MIPs targeting 100% of the known *Klebsiella pneumoniae* carbapenemase (KPC) genes present in the Comprehensive Antibiotic Resistance Database (CARD) to show the utility of 16S classification coupled with AR detection^21^. Evaluation of these probes included testing the 16S probeset together with KPC probes against previously isolated KPC-containing *P. aeruginosa* and *E. cloacae* blood culture samples. Reads mapping to a curated database amalgamating 16S and 19 KPC genes from the CARD database showed the presence of KPC genes in all three replicates for each organism (Fig. 4). An R^2^ value greater than 0.9 was seen when comparing the percentage of mapped 16S sequencing reads in the presence or absence of the KPC probes indicating marginal if any negative effect from their addition (Supplementary Figure S2).

### Performance of 16S and KPC MIPs on mock clinical samples

Finally, we performed a mock clinical analysis of the optimized protocol to access performance using 32 strains chosen from the Food and Drug Administration - Center for Disease Control (FDA-CDC) antimicrobial isolate panel, FDA- Database for Reference Grade Microbial Sequences (FDA-ARGOS), and other reference isolates within the Unified Culture Collection (UCC) (Supplementary Table S2). We designed mock clinical samples to mimic clinical blood cultures utilizing the highest blood-to-culture ratio allowed by the BACTEC FX blood culture system. CFU/ml counts on flagged positive bottles were within a 10^7^–10^9^ range (Supplementary Table S2). Sequencing results for all 31 positive bottles resulted in the detection of 16S reads above an organism negative control blood culture (Table 2). One strain was blood culture negative and was not processed further. Genus level concordance was 96.7% using our optimized adjudication method. Of the 31 blood cultures, only one strain was misidentified: *Klebsiella oxytoca* was identified as the genera *Enterobacter*. To clarify this misidentification, a *de novo* assembly was performed on the sequencing reads, producing three contigs with a sequencing coverage >15,000×. *Klebsiella oxytoca* was not represented in the top 10 hits for any contig when BLAST analysis was performed, indicting potential sample misclassification or contamination of lab stock. If removed from the analysis on the basis of erroneous identification at the stock level, the genus level concordance rate for the other 30 flagged positive blood cultures was 100% (Table 2).

**Table 2:** Taxonomic classification of 16S sequencing reads and detection of KPC genes from mock clinical blood culture samples.

Input Organism	AR Gene	KPC reads	*	Hit 1	%		Hit 2	%		Hit 3	%
*K. pneumoniae*	KPC-3	+	**3**	*K. pneumoniae S000021704*	55.99	**3**	*K. pneumoniae S000387414*	23.51	**3**	*K. variicola*	20.50
*C. freundii*	KPC-2	+	**3**	*C. freundii*	100	**2**	*R. ornithinolytica*	22.4	**2**	*C. murliniae*	21.7
*S. marcescenes*	SME	none	**3**	*S. nematodiphila*	40.41	**3**	*S. marcescens*	38.53	**3**	*S. ureilytica*	21.06
*S. senftenberg*	NDM	none	**3**	*S. enterica S001291914*	100	**2**	S. enterica S004064844	32.6	**2**	S. enterica S000926448	21
*K. ascorbata*	KPC	+	**3**	*K. ascorbata*	84.30	**3**	*K. cryocrescens*	15.70	**2**	*C. freundii*	75.40
*K. oxytoca*	KPC	+	**2**	*E. kobei*	31.37	**2**	*E. ludwigii*	28.61	**2**	*E. asburiae*	22.30
*E. cloacae*	VIM	none	**3**	*E. xiangfangensis*	51.23	**3**	*E. cloacae*	48.77	**2**	E. asburiae	72.9
*P. mirabilis*	KPC	+	**3**	*P. mirabilis*	100	**2**	*P. vulgaris*	50.45	**2**	*P. penneri*	49.54
*E. aerogenes*	IMP	none	**3**	*E. aerogenes*	60.83	**3**	*R. planticola*	39.17	**2**	*R. ornithinolytica*	73.07
*K. pneumoniae*	VIM	none	**2**	*K. pneumoniae S000021704*	44.36	**2**	*K. variicola*	22.84	**2**	*K. pneumoniae S000003514*	16.84
*E. coli*	NDM	none	**3**	*E/S. fergusonii*	50.00	**3**	*E/S. flexneri*	50.00	**2**	*E/S. albertii*	43.76
*K. pneumoniae*	KPC-3	+	**3**	*K. pneumoniae S000021704*	55.96	**3**	*K. pneumoniae S000387414*	24.60	**3**	*K. variicola*	19.44
*S. capitus*	unknown	none	**3**	*S. capitis S000414713*	52.03	**3**	*S. caprae*	47.97	**2**	*S. capitis S000381984*	39.58
*C. indologenes*	unknown	none	**2**	*C. ureilyticum*	26.96	**2**	*C. tructae*	26.38	**2**	*C. lactis*	26.20
*S. lugdenensis*	unknown	none	**3**	*S. lugdunensis*	100	**1**	*S. condimenti*	17.72	**1**	*S. carnosus*	17.34
*S. simulans*	unknown	none	**3**	*S. simulans*	100	**1**	*S. cohnii*	56.59	**1**	*S. argenteus*	8.05
*P. multocida*	unknown	none	**3**	*P. multocida S000390827*	100	**1**	*P. multocida S000390826*	35.74	**1**	*P. multocida S000390828*	32.85
*Y. enterocolitica*	unknown	none	**3**	*Y. enterocolitica*	100	**1**	*Y. massiliensis*	32.59	**1**	*C. gillenii*	32.51
*C. pauculus*	unknown	none	**1**	*C. basilensis*	66.73	**1**	*B. jiangsuensis*	20.52	**1**	*C. pauculus*	6.96
*B. cepecia*	unknown	none	**1**	*B. ambifaria*	41.26	**1**	*B. anthina*	41.76	**1**	*B. lata*	3.49
*P. multocida*	unknown	none	**3**	*P. multocida*	100	**1**	*H. influenzae*	59.4	**1**	*H. felis*	40.6
*S. pyogenes*	unknown	none	**3**	*S. pyogenes*	100	**1**	*S. gordonii*	40.09	**1**	*S. porcorum*	29.45
*A. caviae*	unknown	none	**3**	*A. caviae*	54.01	**3**	*A. taiwanensis*	29.33	**3**	*A. dhakensis*	16.66
*A. baumannii*	OXA-72	none	**3**	*A. baumannii*	100	**2**	*A. venetianus*	63.49	**2**	*A. rudis*	28.72
*E. cloacae*	KPC-3/TEM-1	+	**3**	*E. cloacae*	61.68	**3**	*E. xiangfangensis*	38.32	**2**	*E. asburiae*	53.79
*E. coli*	KPC-3/TEM-1	+	**3**	*E/S. fergusonii*	50.06	**3**	*E/S. flexneri*	49.94	**2**	*E/S. albertii*	43.50
*P. aeruginosa*	KPC	+	**3**	*P. aeruginosa*	100	**1**	*P. stutzeri*	6.58	**1**	*P. nitroreducesn*	6.49
*S. marcescenes*	SME	none	**3**	*S. ureilytica*	100	**2**	*S. nematodiphila*	35.79	**2**	*S. marcescens*	33.30
*P. mirabilis*	NDM	none	**3**	*P. mirabilis*	100	**2**	*P. penneri*	51.31	**2**	*P. vulgaris*	48.60
*K. pneumoniae*	KPC-3	+	**2**	*K. pneumoniae S000003514*	33.59	**2**	*K. variicola*	32.58	**2**	*K. pneumoniae S000387414*	23.79
*K. pneumoniae*	KPC-3	+	**3**	*K. pneumoniae S000021704*	56.25	**3**	*K. pneumoniae S000387414*	23.90	**3**	*K. variicola*	19.85

*Bold indicate number of databases a reference species hit.

Species level concordance was 80% with 24 of the 30 flagged positive cultures being classified correctly (Table 2). This percentage takes into account the classification of *E. coli* as part of the *Escherichia/Shigella fergusonii/flexneri* complex^7^. Concordant species were identified within the top 3 hits in 93% of samples. Issues, such as the low taxonomic resolution for *C. pauculus* and *B. cepecia*, likely resulted from only one variable region being captured. In fact, only five of the mock clinical samples tested had reference species that mapped to less than three variable regions. Of those, the lack of multiple variable regions sequenced resulted in 3/5 being not classified correctly. Impressively, the sensitivity and specificity for KPC gene detection among the isolates was 100% with 11/11 true positives and 9/9 true negatives being correctly called (Table 2). Organisms where AR profiles were unknown were not included in these percentages.

## Discussion

The ability to identify etiologic agents by NGS is quickly becoming a reality for clinical laboratories^22,23^. Simple reference-based genome mapping facilitates identification from metagenomic sequencing of primary samples; however, the etiologic agent to host sequence ratio will always be relatively low for unprocessed clinical samples. This fact limits simultaneous sample multiplexing, lowers throughput, and increases costs of applying NGS assays. Low sequence depth also limits coverage and detection of desirable targets such as AR or virulence genes. Targeted sequencing allows higher coverage for these regions, offering the opportunity to both identify targets and characterize secondary attributes impactful to patient diagnosis.

Several targeted enrichment strategies exist for upfront amplification. Here, we focused on developing a MIP probeset for the enrichment of 16S gene sequences while improving the workflow for routine use and decreased time-to-answer. To address these goals, we combined the hybridization, “gap fill”, and ligation steps to reduce protocol complexity. We also decreased hybridization times to improve time-to-answer. However, these changes could affect the high-order multiplexabilty of MIP pools by negatively impacting capture efficiency. Long hybridization times are a hallmark of numerous hybridization-based techniques including microarrays, MIPs, xGEN Lockdown Probes, and NanoString technologies^24–26^. Overnight hybridizations were necessary to ensure target capture for less efficient probes and to increase specificity by allowing non-target molecules time to dissociate^24^. Here, the optimized hybridization time efficiently captured the targeted sequence; however, we cannot rule out that MIP capture may be impacted by this reduction when we expand the probe panel further. Future assessments of probe additions will resolve this.

The MIP assay had comparable 16S sequence detection to the BactQuant assay, a real-time qPCR 16S gene assay^4^, demonstrating its effectiveness as a molecular tool. Both molecular assays showed reproducible detection at 10^5^ CFU/ml. This limit of detection is well within the average CFU/ml range of 10^7^–10^8^ seen for a flagged positive culture using the BACTEC FX blood culture system. All 31 flagged positive blood culture bottles tested showed positive for 16S sequencing reads. However, for direct detection from primary clinical samples, further LOD improvements would be needed as some intracellular bacterial pathogens can titer to 10^1^ CFU/ml or lower in whole blood^5^. Several conditions could contribute to higher LODs with inefficiencies in extraction likely causing the largest loss of target nucleic acid. Automated extraction methods have multiple clinical benefits such as ease-of-use, time-to-answer, and reproducibility. However, these techniques have known decreases in extraction efficiency compared to manual workflows^27^. Loss of material or degradation of product may have also resulted from an extended mechanical and chemical cell disruption prior to extraction. Bead beating was specifically necessary to ensure extraction and detection from Gram positive organisms such as *B. anthracis*, *S. aureus*, and *E. faecium*. Lastly, carryover inhibitors from blood contaminants may have impacted polymerase or ligase efficiency, thereby potentially affecting 16S sequence capture^4^. Overall, bacterial sample processing in general will need to be solidified before a finalized validated protocol could be established for clinical use.

Bacterial taxonomic classification from 16S gene sequences remains complicated. Full length 16S sequences have the highest levels of taxonomic resolution; however, MIPs capture and amplify only short informative regions requiring several probes to sequence multiple regions. Online tools such as BLAST^28^ and the RDP classifier^29^ were not suitable for ranking reads from multiple separate variable regions. RDP classifier assigns each read a particular taxonomic rank weighting reads that cannot adequately be resolved equally to those that can. For instance, variable regions V6 and V7 of the *Enterobacteriaceae* family have significant intra-genera conservation comparatively to V3; however, using the RDP classifier, each of these regions are weighted similarly^30,31^. Similarly, *de novo* assembly of reads prior to “best hit” BLAST analysis resulted in multiple hits with high sequence identity and low E-values, thus resulting in convoluted identification calls. To mitigate all of these issues, we created a curated reference database composed of the three 16S sequence regions containing V1 and V2, V3, and V6 and V7 from medically relevant genera downloaded from the RDP database. This allowed references that had sequencing reads present in all three regions to be weighted resulting in a high concordance between input etiologic agent and reference call. In fact, this classification method allowed discrimination of mock clinical strains selected from the FDA-CDC Isolate database, which is mostly composed of members from the *Enterobacteriaceae* family. After the analysis, our study showed a genus and species level concordance of 100% and 80% respectively, which is comparable to studies using full 16S sequences^7,32^. Most of the misidentifications, such as *S. marcescenes* as *S. nematodiphila* or *E. cloacae* as *E. xiangfangensis*, were not prevalent human pathogens and could be excluded from analysis. Using this method, speciation of mono-infections like blood cultures was proven to be effective; however, taxonomic resolution of co-infections or complex samples such as wound infections may be difficult to elucidate. Since each variable region is captured and amplified independently, it would be difficult to resolve distinct species if several members of the same genus or family are present. Probes may also bind variable regions of certain species with varying efficiencies due to mutations within the conserved binding site, thus leading to a misrepresentation of mapping percentages. In these instances, the classic 16S amplicon pipeline including clustering sequencing reads into Operational Taxonomic Units (OTUs) combined with a classifier such as RDP could be used, albeit with a cost in taxonomic resolution^33^.

An inherent flaw associated with taxonomic classification using 16S sequences is the inability to resolve species with highly homologous 16S sequences. This is demonstrated in *Y. pestis* and *S. aureus* where strains could not be distinguished from *Y. pseudotuberculosis* and *S. argenteus*. In these instances, MIPs targeting other genomic elements, such as *rpoB* or SNPs, could be used for higher taxonomic resolution including strain determination as demonstrated for *B. anthracis* during the Amerithrax investigation^34^. Unfortunately, the number of targets required to classify all organisms down to this resolution would be not feasible within the current effort. However, probes could be incorporated contingent on the desired diagnostic answer in future efforts. Fortunately MIP technology lends itself to adaptability due in part to the digestion of spurious linear amplicons caused by probe cross-talk^14^. We demonstrated this adaptability, albeit on a small scale, with the addition of the KPC probes to the 16S pool. While this addition showed no impact on overall assay performance, future probe additions would still require bridging studies to ensure new probes are not detrimental to assay performance.

Operationally, MIPs have a similar cost and design structure to other targeted amplification systems such as multiplex PCR. Similar to PCR primers, the target-complementary ends of MIPs have similar design constraints including length, melting temperature, and GC content. Capture region size needs to be considered as MIP efficiency is dependent on backbone length and therefore should be kept consistent among probes^16^. Uniformity in complex GC-rich capture regions should also be kept consistent to ensure effective probe capture. MIPs have a higher upfront cost than PCR, mostly associated with probe prices; however, working concentrations are significantly lower than that of primers and long single-stranded probes are getting progressively cheaper as oligonucleotide synthesis technologies improve. MIPs use affordable reagents such as polymerases, ligases, and restriction enzymes, which do not add greatly to the overall cost of the reaction. Most importantly, PCR and MIPs produce identical products, double-stranded amplicons, resulting in detection by analogous downstream diagnostic technologies.

Unfortunately, library preparation including, indexing, cleanup, and normalization still takes several hours depending on the platform. Similarly, sequencing time is platform contingent, potentially yielding a time-to-answer of days as opposed to hours. However, the advent of new sequencers such as the Illumina MiniSeq and the Ion S5 are pushing the threshold of single day time-to-answer results, making massively parallel sequencing technologies for clinical use a possibility. Single molecule real time sequencers, like the PacBio and MinION, can produce full length 16S sequence and offer the potential to identify etiologic agents in real-time; however each system has caveats, for example high error rates for the MinION nanopore sequencer^35^ or large instrument footprint and initial investment cost for the PacBio. Regardless of the platform used, 16S genes will need to be amplified prior to sequencing to improve signal-to-noise over host background unless being performed from culture. MIPs represent a potential solution for this issue, allowing for the capture of multiple gene regions for species level taxonomic identification and characterization and providing a step forward towards the application of NGS in the clinical setting.

## Material and Methods

### Strains used, DNA preparation, and CFU estimation

Bacterial strains used in this study are included in Supplementary Table S2. For optimization experiments DNAs were extracted and purified using the Qiagen EZ1 DNA Tissue kit (Qiagen, Valencia, Ca) according to the manufacturer’s instructions. DNA concentration was quantified utilizing Qubit dsDNA BR and HS assay kits (Life Technologies, Carlsbad, CA). For all other experiments to determine CFU/ml bacterial cultures were grown overnight in tryptic soy broth (Thermo Fisher Scientific, Waltham, MA), concentrated by centrifugation and optical density of 2-fold serial dilutions was measured with a Tecan 200 PRO series (Mannedorf, Switzerland). Cells were plated directly from these stocks on sheep’s blood agar plates (Thermo Fisher Scientific), grown overnight at 37 °C, and counted for colony formation. A linear optical density range for each organism was determined and used to determine CFU/ml in future experiments. For analytical analysis input CFU’s were resuspended in 1 ml of BACTC Standard/10 aerobic/F culture spiked with whole blood (BioreclamationIVT, Baltimore, MD) at a 1:4 ratio and 10 fold serially diluted. For mock clinical samples, a colony was suspended in 40 mls of BACTC Standard/10 aerobic/F culture spiked with whole blood at a 1:4 ratio. Bottles were then cultured in a BACTEC FX40 (Thermo Fisher Scientific) overnight. 50 µl lysozyme (100 mg/ml) and 10 μl of mutanolysin (10,000 U/ml) were added to each 1 ml sample and incubated at 37 °C for 30 minutes. Samples were then bead beat for 5 minutes with 100 μl of 0.5 µm beads. 200 µl of this was removed and DNA was extracted as described above according to manufacturer’s protocols.

### MIP design and protocol

MIP complementary 16S probe arms were designed utilizing CLC Genomic Workbench (CLC Bio, Cambridge, MA) and AlleleID 7.73 (PREMIER Biosoft, Palo Alto, CA). Primers were designed targeting the conserved regions flanking variable regions 1, 2, 3, 6 and 7 of the 16S based on their ability to distinguish pathogenic bacteria^9^. For KPC gene detection sequences were downloaded from the Comprehensive Antibiotic Resistance Database (CARD) and aligned using Clustal W. Conserved regions were evaluated and probe arms were designed as previously described. Probe arms were flanked by a set of universal primers previously characterized^36^ and a lambda based common backbone^16^. Probes were synthesized by Integrated DNA Technologies (IDT, Coralville, IA). Complimentary probe arms, universal primers, and linker backbone are represented in Supplementary Table S1.

Probes were re-suspended in water and pooled in equimolar amounts at concentrations indicated. A total of 8 probes were combined and used as a master probe mix for 16S detection. Two KPC genes were later added for a 10 probe mix pool. The MIP protocol was performed as follows: Reaction mixtures contained 1× Phusion high-fidelity PCR master mix with HF buffer (New England Biolabs, Ipswich, MA), 10 units of Ampligase (Epicentre, Madison, WI), 500 μM Nicotinamide adenine dinucleotide (Sigma-Aldrich, St. Louis, MO), indicated concentration of MIP pool, and indicated amounts of DNA with water in a final volume of 10 μl. The reaction mixture was incubated at 98 °C for 3 minutes, ramped to 55 °C (0.1 °C/sec) and held for 60 minutes, 72 °C for 15 minutes, and finally held at 4 °C indefinitely. For the exonuclease reaction 20 units of exonuclease I (NEB), 25 units of exonuclease III (NEB), and water were added to the reaction mixture up to a final volume to 11.5 μl. The mixture was then incubated at 37 °C for 30 minutes, 80 °C for 20 minutes, and held at 4 °C indefinitely. To amplify the capture region 1× Phusion high-fidelity PCR master mix with HF buffer was added along with 0.5 μM of forward and reverse universal primers and water for a final reaction mixture volume of 20 ul. The reaction mixture was amplified as follows: 98 °C for 3 minutes, then 98 °C for 10 seconds, 60 °C for 30 seconds, and 72 °C for 15 seconds for 40 cycles, 72 °C for 5 minutes and held at 4 °C indefinitely. The amplicons were purified utilizing Agencourt AMPure XP beads (Beckman Coulter, Pasadena, Ca) per the manufactures protocol with a bead ration of 0.7×. For optimization experiments samples amplicon concentrations were measured with the LabChip GX Touch HT using the high sensitivity kit (PerkinElmer, Waltham, MA) using a 300–600 bp region for analysis.

### Database Curation

The Ribosomal Database Project (RDP) was used to curate a reference database composed of isolates of type strains greater than 1200 base pairs of good quality^29^. Genera of medically relevant pathogens were selected and all species in those genera were included. A final reference database composed of 3,426 sequences encompassing 88 genera and 3,069 species was made (Supplementary Table 3)^7^. Based on MIP target capture three databases composed of V1V2, V3, and V6V7 respectively were isolated from each reference and used for reference based read mapping. For each species, references with 100% nucleotide similarity were collated into one reference. For AR genes, 19 KPC genes from the CARD database were included in the curated database^21^.

### Sequencing and analysis

Library preparation was performed with Nextera dual indexes (Illumina, San Diego, CA) and the Kapa Biosystems Library Amplification Kit (Kapa Biosystems, Wilmington, MA). Briefly the reaction mixture contained 1× HotStart mix, 3 µl each of Nextera Index Primer N7XXX and S5XX, 3 µl primer mix, and 6 µl of MIP reaction amplicon for a final volume of 30 μl. The reaction was then amplified as follows: 72 °C for 3 minutes, 98 °C for 30 seconds, then 98 °C for 10 seconds, 63 °C for 30 seconds, and 72 °C for 3 minutes for 25 cycles, 72 °C for 1 minutes and held at 4 °C indefinitely. The amplicons were purified utilizing Agencourt AMPure XP beads (Beckman Coulter, Pasadena, Ca) per the manufactures protocol with a 0.5× mixture of bead to sample volume. Samples were quantified with the the LabChip GX Touch HT using the high sensitivity kit (PerkinElmer, Waltham, MA). Samples were then pooled based on total concentration. Adaptor ligation confirmation and concentration of the pool was performed using the KAPA library quantification kit (Kapa Biosystems). Amplicons were sequenced using the MiSeq platform (Illumina) using the v2 500 cycle sequencing kit. For Fig. 2, data was analyzed from three separate sequencing reactions each containing 75 pooled samples. Extracted DNA from this sample set was re-tested for Fig. 4 using a probe pool including 16S and KPC MIPs and run on a separate sequencing reaction. Mock clinical samples were all pooled and evaluated using one sequencing reaction.

Analysis was performed using CLC genomic workbench. Paired end reads were merged and adaptor trimmed using the universal sequences CGTTGTTACCGACTGGATTATTACC and TCCGCATACCAGTTGTTGTCG a quality score 0.05 and sequence length of >100 bp. A stringent referenced based mapping of sequencing reads to the RDP reference databases V1V2, V3, and V6 was used. Mapping settings were as follows: mismatch cost of 10, insertion cost of 3, deletion cost of 3, insertion open cost of 6, insertion extend cost of 1, deletion open cost of 6, deletion extend cost of 1, length fraction of 0.5, and similarity fraction of 0.9. Total numbers of mapped reads and % of mapped reads to merged paired end reads before trim were used. GraphPad Prism v7.01 and JMP Genomics v8.1 were used for statistical analysis and graphing.

### Real-time PCR analysis

Real-time PCR analysis was performed utilizing the BactQuant qPCR 16S assay^4^. Forward Primer (5′- CCTACGGGDGGCWGCA-3′), reverse primer (5′- GGACTACHVGGGTMTCTAATC -3′) and probe ((6FAM) 5′-CAGCAGCCGCGGTA-3′ (MGBNFQ)) were used at 1.8 μM and 0.225 μM concentrations respectively with 1× Platinum Quantitative PCR SuperMix UDG (Thermo Fisher Scientific) in a final volume of 10 μl. The reaction mixture was amplified as follows: 50 °C for 3 minutes, 95 °C for 10 minutes, then 40 cycles of 95 °C for 15 seconds, and 60 °C for 1 min. Assays were run on the Roche LightCycler 480 (Roche Applied Science, Indianapolis, IN) and a single fluorescence read was taken at the end of each 60 °C step. Absolute quantification analysis using the 2^nd^ derivative quantification method was used on each sample. Samples with no Cq value were given a cutoff value of 40.

### Data Availability

The datasets generated during and/or analyzed during the current study are available from the corresponding author on reasonable request.

## Electronic supplementary material

Supplementary Information
