# Supplementary material for: Detection of 16S rRNA and KPC Genes from Complex Matrix Utilizing a Molecular Inversion Probe Assay for Next-Generation Sequencing
Source: Sci Rep. 2018 Feb 1;8:2028. doi: 10.1038/s41598-018-19501-z (PMC5794912; doi:10.1038/s41598-018-19501-z)
Supplement: Supplementary file 1 — Supplementary Information [file 41598_2018_19501_MOESM1_ESM.pdf]

Detection of 16S rRNA and KPC Genes from Complex Matrix Utilizing a Molecular Inversion Probe Assay for Next-Generation Sequencing

Christopher P. Stefan[1], Adrienne T. Hall[1], Timothy D. Minogue[1*]

[1] United States Army Medical Research Institute of Infectious Disease, Diagnostic Systems Division, Fort Detrick, Maryland, 21702, United States of America

christopher.p.stefan.ctr@mail.mil

adrienne.t.hall2.civ@mail.mil

[*]timothy.d.minogue.civ@mail.mil

Supplementary Information

Supplementary Figure S1: Amplicon purity versus concentration and temperature conditions. Pooled 16S MIPs were tested against DNA extracts of representative biothreats and ESKAPE pathogens to optimize target capture conditions including (A) probe concentration and (B) reaction temperature. Amplicon formation was measured after probe circularization and amplification with the universal primer set. Three replicates for each organism at each variable were performed and the percent purity was measured as the concentration of the desired amplicon over total sample concentration.

Supplementary Figure S2. Correlation of the percentage of mapped sequencing reads of the 16S MIP probeset with and without the KPC probes. Pooled 16S MIPS with and without KPC MIPs were tested against DNA extracted from carbapenemase producing (A) *Enterobacter cloacae* and (B) *Pseudomonas aeruginosa* that were serial diluted into blood culture medium. The percentage of mapped reads at each dilution was plotted and Pearson correlation analysis was performed.

Supplementary Figure S1:

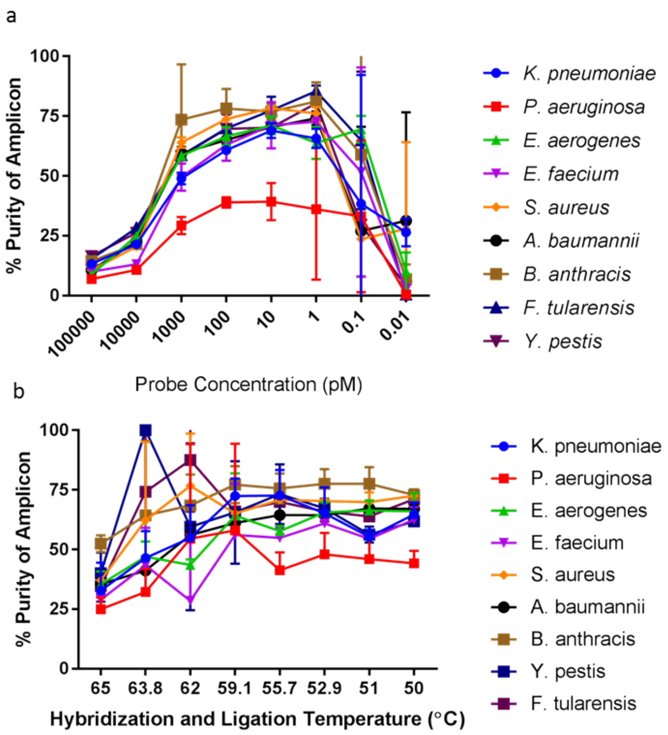

Supplementary Figure S2. Correlation of the percentage of mapped sequencing reads of the 16S MIP probeset with and without the KPC probes

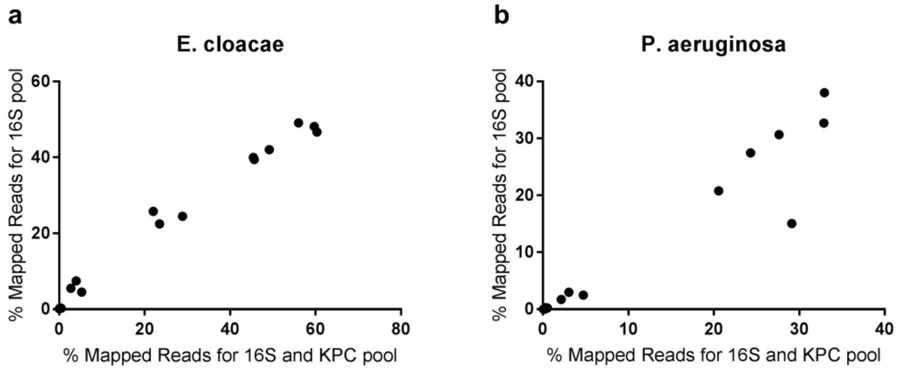

Pearson r	
r	0.9939
95% confidence interval	0.9856 to 0.9974
R squared	0.9878

Pearson r	
r	0.9657
95% confidence interval	0.9211 to 0.9853
R squared	0.9326

Supplementary Table S1: Primers and Probes used in this study

Name	Sequence
V2	/5'Phos/ttactcacccgtycgccrcGGTAATAATCCAGTCGGTAACAACGAACGGTACGCTGAGGGCGGAAAAAATCGTCGGGGACATTGTAAAGGCGGCGAGCGCGGCTTTTCCGCGCCAGCGTGAAAGCAGTGTGGACTGGCCGTCAGGTACTCCGCATACCAGTTGTTGTCGctgctgcctcccgtaggag
V1V2	/5'Phos/ctgagccakgatcaaactctGGTAATAATCCAGTCGGTAACAACGAACGGTACGCTGAGGGCGGAAAAAATCGTCGGGGACATTGTAAAGGCGGCGAGCGCGGCTTTTCCGCGCCAGCGTGAAAGCAGTGTGGACTGGCCGTCAGGTACTCCGCATACCAGTTGTTGTCGctgctgcctcccgtaggag
V3-1	/5'Phos/ctgctgcctcccgtaggagGGTAATAATCCAGTCGGTAACAACGAACGGTACGCTGAGGGCGGAAAAAATCGTCGGGGACATTGTAAAGGCGGCGAGCGCGGCTTTTCCGCGCCAGCGTGAAAGCAGTGTGGACTGGCCGTCAGGTACTCCGCATACCAGTTGTTGTCGgtattaccgcrgctgctgg
V3-2	/5'Phos/gctgcctcccgtaggagtGGTAATAATCCAGTCGGTAACAACGAACGGTACGCTGAGGGCGGAAAAAATCGTCGGGGACATTGTAAAGGCGGCGAGCGCGGCTTTTCCGCGCCAGCGTGAAAGCAGTGTGGACTGGCCGTCAGGTACTCCGCATACCAGTTGTTGTCGgtattaccgcrgctgctgg
V6V7-1	/5'Phos/ggtaaggttyytcgcgttgcGGTAATAATCCAGTCGGTAACAACGAACGGTACGCTGAGGGCGGAAAAAATCGTCGGGGACATTGTAAAGGCGGCGAGCGCGGCTTTTCCGCGCCAGCGTGAAAGCAGTGTGGACTGGCCGTCAGGTACTCCGCATACCAGTTGTTGTCGgacgtcrtccccaccttcc
V6V7-2	/5'Phos/ggtaaggttyytcgcgttgcGGTAATAATCCAGTCGGTAACAACGAACGGTACGCTGAGGGCGGAAAAAATCGTCGGGGACATTGTAAAGGCGGCGAGCGCGGCTTTTCCGCGCCAGCGTGAAAGCAGTGTGGACTGGCCGTCAGGTACTCCGCATACCAGTTGTTGTCGttgacgtcrtccccaccttcc
V6-1	/5'Phos/gcgggbccccgtcaattcGGTAATAATCCAGTCGGTAACAACGAACGGTACGCTGAGGGCGGAAAAAATCGTCGGGGACATTGTAAAGGCGGCGAGCGCGGCTTTTCCGCGCCAGCGTGAAAGCAGTGTGGACTGGCCGTCAGGTACTCCGCATACCAGTTGTTGTCGgagctgacgacarccatgc
V6-2	/5'Phos/ccccgtcaattcmtttragtttGGTAATAATCCAGTCGGTAACAACGAACGGTACGCTGAGGGCGGAAAAAATCGTCGGGGACATTGTAAAGGCGGCGAGCGCGGCTTTTCCGCGCCAGCGTGAAAGCAGTGTGGACTGGCCGTCAGGTACTCCGCATACCAGTTGTTGTCGagggttgcgctcgttg
KPC-1	/5'Phos/gagccgccaaagtcctgttGGTAATAATCCAGTCGGTAACAACGAACGGTACGCTGAGGGCGGAAAAAATCGTCGGGGACATTGTAAAGGCGGCGAGCGCGGCTTTTCCGCGCCAGCGTGAAAGCAGTGTGGACTGGCCGTCAGGTACTCCGCATACCAGTTGTTGTCGaattggcggcggcgtta
KPC-2	/5'Phos/ctgctgctggctgcgagGGTAATAATCCAGTCGGTAACAACGAACGGTACGCTGAGGGCGGAAAAAATCGTCGGGGACATTGTAAAGGCGGCGAGCGCGGCTTTTCCGCGCCAGCGTGAAAGCAGTGTGGACTGGCCGTCAGGTACTCCGCATACCAGTTGTTGTCGccgcccaactccttcagc
Universal-1	**TCGTCGGCAGCGTCAGATGTGTATAAGAGACAG**NNNNCGTTGTTACCGACTGGATTATTACC
Universal-2	**GTCTCGTGGGCTCGGAGATGTGTATAAGAGACAG**NNNNTCCGCATACCAGTTGTTGTCG

BactQuant-F	CCTACGGGDGGCWGCA
BactQuant-R	GGACTACHVGGGTMTCTAATC
BactQuant-P	(6FAM) 5′-CAGCAGCCGCGGTA-3′ (MGBNFQ)

* Italicized nucleotides represent gene specific sequences
** Underlined nucleotides represent universal primer sequence
*** Bold nucleotides represent nextera adaptors

Supplementary Table S2: Strains used in study

MIP optimization		Blood Culture Dilution Series	
Organism	Strain/Ascension	Organism	Strain/Ascension
Bacillus anthracis	ΔANR BACI355	*Bacillus anthracis*	ΔANR BACI355
Yersinia pestis	KIM5 YERS052	*Yersinia pestis*	KIM5 YERS052
Francisella tularensis	LVS	*Francisella tularensis*	LVS
Enterococcus faecium	10857-1	*Staphylococcus aureus*	FDAARGOS169
Staphylococcus aureus	10778-1	*Enterococcus faecium*	EFUAA714
Klebsiella pneumoniae	12705-1	*Pseudomonas aeruginosa*	SAMN04014931
Acinetobacter baumannii	10737-1	*Klebsiella pneumoniae*	SAMN04014885
Pseudomonas aeruginosa	10712-2	*Acinetobacter baumannii*	SAMN04014886
Enterobacter aerogenes	1311-8-72	*Enterobacter cloacae*	SAMN04014977

Mock Clinical					
FDA-CDC Isolate bank	Strain/Ascension	CFU/ml for Flagged +	FDA-Argos	Strain/Ascension	CFU/ml for Flagged +
Klebsiella pneumoniae	SAMN04014953	1.13E+09	*Staphylococcus capitus*	CNH809	2.70E+07
Citrobacter freundii	SAMN04014957	1.32E+09	*Chryseobacterium indologenes*	CNH811	1.14E+09
Serratia marcescenes	SAMN04014962	2.65E+09	*Staphylococcus lugdenensis*	CNH815	1.15E+08
Salmonella senftenberg	SAMN04014968	1.35E+09	*Staphylococcus simulans*	CNH818	3.25E+07
Kluyvera ascorbata	SAMN04014985	1.1E+09	*Pasturella multocida*	CNH821	3.00E+09
Klebsiella oxytoca	SAMN04014988	1.4E+09	*Yersinia enterocolitica*	CNH911	2.34E+09
Enterobacter cloacae	SAMN04014995	1.39E+09	*Cupriavidus pauculus*	CNH1000	1.80E+07
Proteus mirabilis	SAMN04014996	1.4E+09	*Burkholderia cepecia*	CNH1001	5.75E+08
Enterobacter aerogenes	SAMN04015002	1.74E+09	*Pasturella multocida*	ULK41	1.67E+09
Klebsiella pneumoniae	SAMN04014976	9.95E+08	*Streptococcus pyogenes*	CNH1302	1.42E+08
Escherichia coli	SAMN04014990	1.44E+09	*Aeromonas caviae*	ULK5	1.61E+09
Klebsiella pneumoniae	SAMN04014954	1.34E+09	*Bordetella bronchioseptica*	ULK13	0.00E+00
Acinetobacter baumannii	SAMN04014876	1.07E+08			
Enterobacter cloacae	SAMN04014894	1.56E+09			
Escherichia coli	SAMN04014902	2.65E+08			
Pseudomonas aeruginosa	SAMN04014931	6.65E+08			
Serratia marcescenes	SAMN04014964	1.44E+09			
Proteus mirabilis	SAMN04015000	4.7E+08			
Klebsiella pneumoniae	SAMN04014958	1.02E+09			
Klebsiella pneumoniae	SAMN04014956	9.7E+08			

Supplementary Table S3: Genera included in Reference Database

Abiotrophia	*Campylobacter*	*Francisella*	*Orientia*	*Streptococcus*
Acidovorax	*Capnocytophaga*	*Fusobacterium*	*Pandoraea*	*Streptomyces*
Acinetobacter	*Chlamydia*	*Gemella*	*Pasteurella*	*Treponema*
Actinobacillus	*Chlamydophila*	*Gordonia*	*Peptoniphilus*	*Tropheryma*
Actinomadura	*Chryseobacterium*	*Haemophilus*	*Peptostreptococcus*	*Ureaplasma*
Actinomyces	*Citrobacter*	*Helicobacter*	*Plesiomonas*	*Veillonella*
Aerococcus	*Clostridium*	*Kingella*	*Porphyromonas*	*Vibrio*
Aeromonas	*Comamonas*	*Klebsiella*	*Prevotella*	*Yersinia*
Anaerococcus	*Coxiella*	*Kluyvera*	*Propionibacterium*	
Anaplasma	*Cupriavidus*	*Lactobacillus*	*Proteus*	
Arcobacter	*Delftia*	*Leclercia*	*Pseudomonas*	
Bacillus	*Dermabacter*	*Legionella*	*Ralstonia*	
Bacteroides	*Ehrlichia*	*Leptospira*	*Raoultella*	
Bartonella	*Eikenella*	*Listeria*	*Rhodococcus*	
Bordetella	*Enterobacter*	*Micrococcus*	*Rickettsia*	
Borrelia	*Enterococcus*	*Moraxella*	*Salmonella*	
Brachyspira	*Erysipelothrix*	*Mycobacterium*	*Serratia*	
Brevundimonas	*Escherichia/Shigella*	*Mycoplasma*	*Shigella*	
Brucella	*Facklamia*	*Neisseria*	*Staphylococcus*	
Burkholderia	*Finegoldia*	*Nocardia*	*Stenotrophomonas*	

*All species within each genus were included in the curated databases
